# Data on whole genome sequencing of the oomycete *Pythium insidiosum* strain CBS 101555 from a horse with pythiosis in Brazil

**DOI:** 10.1186/s13104-018-3968-3

**Published:** 2018-12-11

**Authors:** Theerapong Krajaejun, Weerayuth Kittichotirat, Preecha Patumcharoenpol, Thidarat Rujirawat, Tassanee Lohnoo, Wanta Yingyong

**Affiliations:** 1 Department of Pathology, Faculty of Medicine, Ramathibodi Hospital, Mahidol University, Bangkok, Thailand; 2 Systems Biology and Bioinformatics Research Group, Pilot Plant Development and Training Institute, King Mongkut’s University of Technology Thonburi, Bangkhuntien, Bangkok, Thailand; 3 Research Center, Faculty of Medicine, Ramathibodi Hospital, Mahidol University, Bangkok, Thailand

**Keywords:** *Pythium insidiosum*, Pythiosis, Oomycete, Genome, Gene cluster, Sequence variant

## Abstract

**Objectives:**

The oomycete *Pythium insidiosum* infects humans and animals worldwide, and causes the life-threatening condition, called pythosis. Most patients lose infected organs or die from the disease. Comparative genomic analyses of different *P. insidiosum* strains could provide new insights into its pathobiology, and can lead to discovery of an effective treatment method. Several draft genomes of *P. insidiosum* are publicly available: three from Asia (Thailand), and one each from North (the United States) and Central (Costa Rica) Americas. We report another draft genome of *P. insidiosum* isolated from South America (Brazil), to serve as a resource for comprehensive genomic studies.

**Data description:**

In this study, we report genome sequence of the *P. insidiosum* strain CBS 101555, isolated from a horse with pythiosis in Brazil. One paired-end (180-bp insert) library of processed genomic DNA was prepared for Illumina HiSeq 2500-based sequencing. Assembly of raw reads provided genome size of 48.9 Mb, comprising 60,602 contigs. A total of 23,254 genes were predicted and classified into 18,305 homologous gene clusters. Compared with the reference genome (the *P. insidiosum* strain Pi-S), 1,475,337 sequence variants (SNPs and INDELs) were identified in the organism. The genome sequence data has been deposited in DDBJ under the accession numbers BCFP01000001–BCFP01060602.

## Objective

*Pythium insidiosum* is a fungus-like, aquatic, oomycetous microorganism that belongs to the kingdom Straminipila [1]. Microscopic features of *P. insidiosum* resemble that of filamentous fungi. The organism can be divided into three phylogenetical groups, in association with geographical origins: Clade-I strains (North, Central, and South Americas); Clade-II strains (Asia and Australia), and Clade-III strains (Thailand and the United States). In nature, *P. insidiosum* is observed in two forms: mycelium and zoospore (an infective unit) [2]. Several groups of investigators have successfully isolated *P. insidiosum* from swampy areas in Australia, Thailand, the United States, and Brazil [3–6]. While most pathogenic *Pythium* species infects plants, *P. insidiosum* infects humans and animals, and causes the life-threatening disease, called pythosis [7]. Case reports of the *P. insidiosum* infection in humans are almost exclusively from Asia, while that in animals are mainly from North, Central, and South Americas [1, 7]. Diagnosis of pythiosis is difficult. Treatment of this disease is challenging because effective drug and vaccine are lacking. Despite intensive cares are provided, most patients have their infected organs (i.e., eye, arm, leg) removed, and many patients die from the progressive infection [7].

Genome sequence can be used to explore pathobiology of an organism of interest. It is now feasible to sequence the genome of the non-model organism (i.e., *P. insidiosum*) using the next generation sequencing technologies. Comparative genomic analyses of different *P. insidiosum* strains could provide new insights into its biological processes and pathogenesis, which can lead to discovery of a novel method for pathogen control. Five draft genomes of *P. insidiosum* are deposited in the public repositories: three from Asia (Thailand; Clade-II and -III strains), and one each from North (the United States; Clade-I strain) and Central (Costa Rica; Clade-I strain) Americas [8–11]. Here, we report another draft genome data of *P. insidiosum* (Clade-I) isolated from South America (Brazil), as opposed to the other 5 strains (with published genome sequences) isolated from other regions of the world, to serve as a resource for comprehensive genomic studies in the future.

## Data description

The *P. insidiosum* strain CBS 101555, isolated from a granulomatous lesion at the abdomen of a horse with pythiosis living in the southern region of Brazil, was cultured in Sabouraud dextrose broth at 37 °C for 1 week. Hyphal mat was harvested from the culture, and subjected to genomic deoxyribonucleic acid (DNA) extraction, using the conventional extraction method, optimized for *P. insidiosum* [12]. The identity of the strain was checked by single nucleotide polymorphism-based multiplex PCR and sequence homology analysis of the rDNA sequence (Accession number: AB971181) [13, 14]. The obtained genomic DNA was sequenced, using the Illumina next generation sequencing platform, as previously-described [8–10]. Briefly, the genomic DNA was processed to prepare a paired-end (180-bp insert) library for Illumina HiSeq 2500-based sequencing (Yourgene Bioscience, Taiwan). To guarantee read lengths of at least 35 bases, obtained raw reads underwent quality trims by CLC Genomics Workbench (Qiagen). The Cutadapt 1.8.1 [15] was used to remove the adaptor sequences. The resulting genome data contained 34,617,696 raw reads with an average length of 122 bases, providing 4,233,254,451 total bases. Genome assembly, performed by Velvet 1.2.10 [16], showed a total of 60,602 contigs, an average contig length of 806 bases (range 300–30,744), *N*_50_ of 953 bases, and ‘N’ composition of 0.9%. The draft assembled genome size of the organism was 48,855,945 bases. MAKER2 [17] predicted 23,254 genes in the draft genome. Basic Local Alignment Search Tool (BLAST) was used to annotate predicted genes by comparing to the NCBI non-redundant protein database using *E*-value cut off 10^−6^. Product description of the best blast hit was used as the product description of the query gene. The genome sequence data has been deposited in the DNA Data Bank of Japan (DDBJ) under the Accession numbers BCFP01000001–BCFP01060602 (Data file 1; Table 1).

**Table 1:** Overview of data files/data sets

Label	Name of data file/data set	File types (file extension)	Data repository and identifier (DOI or accession number)
Data file 1	Whole genome sequence	FASTA	DDBJ (Accession numbers: BCFP01000001–BCFP01060602) (http://getentry.ddbj.nig.ac.jp/)
Data file 2	Gene clusters	MS Excel file (.xlsx)	Mendeley database (10.17632/yjyzx5gk7s.1) (https://data.mendeley.com/datasets/yjyzx5gk7s/1)
Data file 3	Clusters of Orthologous Groups of Proteins (COGs)	MS Excel file (.xlsx)	Mendeley database (10.17632/5rhfd4n37k.1) (https://data.mendeley.com/datasets/5rhfd4n37k/1)
Data file 4	Sequence variants	MS Excel file (.xlsx)	Mendeley database (10.17632/4y8hdw7tb7.1) (https://data.mendeley.com/datasets/4y8hdw7tb7/1)

The 23,254 predicted genes can be classified into 18,305 homologous gene clusters (Data file 2; Table 1), using the method described by Kittichotirat et al. [18] and Rujirawat et al. [19], and the following setting: BLAST *E*-value of 10^−6^, pairwise sequence identity of at least 30%, and pairwise alignment coverage for both query and subject sequences of at least 50%. Based on the BLAST search with *E*-value cut-off of 10^−6^ against the Clusters of Orthologous Groups of Proteins (COGs) database [20, 21], 3288 gene clusters (18%) were assigned to 24 COGs groups, while the rest (15,017 gene clusters [82%]; designated as uncharacterized cluster) did not match any COGs. Details on percentages and frequency of each assigned COGs group were shown in Data file 3 (Table 1).

The obtained draft genome was analysed for sequence variants, by using the Burrows–Wheeler Alignment tool [22]. Approximately, 44% of the processed reads (n = 15,084,792) of the *P. insidiosum* strain CBS 101555 can map the reference genome of the *P. insidiosum* strain Pi-S (the genome size of 53,239,050 bases, comprising 1192 contigs; Accession number BBXB00000000.1) [10]. FreeBayes [23] can identify 1,475,337 sequence variants, including single-nucleotide polymorphisms (SNPs) and insertion/deletion of bases (INDELs), in the genome of the organism (Data file 4; Table 1).

In conclusion, *P. insidiosum* is an understudied pathogen that causes the life-threatening condition, called pythiosis, in humans and animals worldwide. We sequenced the draft genome of the *P. insidiosum* strain CBS 101555, isolated from a pythiosis horse living in the southern region of Brazil. The obtained genome will be a fundamental resource for exploring biology and pathogenesis of this invasive microorganism.

## Limitations

The draft genome was obtained from short-read assembly of one Illumina-based paired-end (180-bp insert) library, without any mate pair library, resulting in as many as 60,602 contigs. The estimated genomic coverage is limited to ~ 87-fold. The mitochondrial genome sequences were not excluded from the nuclear genome assembly.

## Abbreviations

DNA: deoxyribonucleic acid
DDBJ: DNA Data Bank of Japan
BLAST: Basic Local Alignment Search Tool
COGs: Clusters of Orthologous Groups of Proteins
SNP: single-nucleotide polymorphism
INDEL: insertion or deletion of bases
